# Analysis of C4 and the C4 binding protein in the MRL/lpr mouse

**DOI:** 10.1186/ar2320

**Published:** 2007-10-30

**Authors:** Scott E Wenderfer, Kipruto Soimo, Rick A Wetsel, Michael C Braun

**Affiliations:** 1 Center for Immunology and Autoimmune Diseases, Brown Foundation Institute of Molecular Medicine, 1825 Pressler Street, Houston, TX 77030, USA; 2 Pediatric Nephrology, University of Texas, 6431 Fannin Street, Houston, TX 77030, USA

## Abstract

Systemic lupus erythematosus is a complement-mediated autoimmune disease. While genetic deficiencies of classical pathway components lead to an increased risk of developing systemic lupus erythematosus, end organ damage is associated with complement activation and immune complex deposition. The role of classical pathway regulators in systemic lupus erythematosus is unknown. C4 binding protein (C4bp) is a major negative regulator of the classical pathway. In order to study the role of C4bp deficiency in an established murine model of lupus nephritis, mice with a targeted deletion in the gene encoding C4bp were backcrossed into the MRL/lpr genetic background. Compared with control MRL/lpr mice, C4bp knockout MLR/lpr mice had similar mortality and similar degrees of lymphoproliferation. There were no differences in the extent of proteinuria or renal inflammation. Staining for complement proteins and immunoglobulins in the kidneys of diseased mice revealed no significant strain differences. Moreover, there was no difference in autoantibody production or in levels of circulating immune complexes. In comparison with C57BL/6 mice, MRL/lpr mice had depressed C4 levels as early as 3 weeks of age. The absence of C4bp did not impact serum C4 levels or alter classical pathway hemolytic activity. Given that immune complex renal injury in the MRL/lpr mouse is independent of Fc receptors as well as the major negative regulator of the classical pathway, new mechanisms for immune-complex-mediated renal injury need to be considered.

## Introduction

The complement system is an important mediator of tissue injury in systemic lupus erythematosus (SLE) and other immune complex diseases. SLE is characterized by systemic complement activation, autoantibody production, the formation of circulating immune complexes, and the generation of autoreactive lymphocytes associated with multisystem injury, including nephritis, arthritis, serositis, dermatitis, and blood dyscrasias. Lupus nephritis is mediated in part by local deposition of circulating immune complexes and complement activation products. The relationship of complement to the pathogenesis of SLE is a complex one. Genetic deficiencies in the early components of the classical complement pathway (C1 inhibitor, C1q/r/s, C2, or C4) are some of the strongest risk factors for the development of SLE [1]. This is thought to be due to the role of the early classical pathway of complement activation in the clearance of immune complexes and apoptotic cells. Systemic complement activation, however, marked by depression of serum C3 and C4 levels and peripheral deposition of these proteins, is associated with increased disease activity [2,3].

The complement system can be activated by three pathways: the classical pathway and the lectin pathway both require the fourth component of complement (C4), while the alternative pathway is independent of C4. All three pathways activate C3 by forming an enzyme, the C3 convertase, which cleaves C3 generating the C3a anaphylatoxin and the activation product C3b. The product C3b mediates a number of cellular reactions leading to proliferation and cell activation, release of proinflamatory cytokines, increased vascular permeability, cell recruitment, apoptosis, and, ultimately, parenchymal damage [4].

C4 binding protein (C4bp) negatively regulates activation of the classical pathway and the lectin pathway [5-7]. Functionally, C4bp limits complement activation by blocking the formation of and promoting the decay of the classical pathway C3 convertase. It acts via three mechanisms: preventing the formation of the C3 convertase by binding to C4b; accelerating the natural decay of the classical pathway C3 convertase; and as a cofactor for the serine proteinase factor I in the proteolytic inactivation of C4b, which prevents the formation of the C3 convertase. Deficiency of C4bp would be expected to result in increased cleavage of C3 and in increased complement activity in response to classical pathway or lectin pathway activation by immune complex formation, bacterial infections, apoptosis, and other triggering mechanisms.

C4bp is present in human serum at concentrations of approximately 200 mg/l [8]. Human C4bp is synthesized primarily in the liver, and to a lesser degree by activated monocytes [9]. It is an acute phase reactant [10,11], with expression upregulated by proinflammatory cytokines [9,11]. In addition, C4bp protein levels have been shown to be upregulated in SLE [10]. Only one patient with C4bp deficiency has been described [12]. She had levels that were 15–29% of normal with repeated testing by radioimmunodiffusion. The patient presented at age 33 years with recurrent oral and genital ulcers, angioedema, malar rash, photosensitivity, dysuria, undetectable antinuclear antibodies, and normal C1 inhibitor levels. Biopsy of her skin lesions revealed arteriolar vasculitis with perivascular monocytic infiltrates, and increased C3 and IgM staining. The patient was diagnosed with atypical Behcet's disease and was treated with solumedrol and cyclophosphamide. Genotyping was not reported, but her father and her sister were reported to have similarly low serum C4bp levels [13]. There have been no reported cases of C4bp deficiency in patients with SLE.

C4bp belongs to a gene family of structurally related proteins designated the regulators of complement activation. There are three isoforms of C4bp in humans [6]. The predominant form is a 570 kDa glycoprotein composed of seven α chains covalently bound to each other and to one β chain. Other isoforms contain either seven α chains without a β chain or six α chains with one β chain. The α chain is composed of eight complement control protein domains, and the N-terminal three complement control proteins bind C4b [14]. The C-terminus contains a separate domain critical for multimerization. The β chain contains three complement control protein domains.

Human C4bp has been shown to bind other compounds including protein S (β chain), C-reactive protein, serum amyloid protein, soluble CD40 ligand, CD40 (α chain), heparin (α chain), low-density lipoprotein receptor protein (α chain), and several bacterial peptides (α chain) [6,15-21]. Isoforms containing the β chain can also bind to negatively charged phospholipids on the surface of apoptotic cells in a protein-S-dependent manner [22]. All isoforms regulate complement in an equivalent manner, and no binding partner has been shown to modulate C4bp complement regulatory activity.

The structure of murine C4bp differs from its human ortholog. The mouse protein lacks the β chain [23] and the murine α chain lacks two complement control protein domains and four cysteines present in human C4bp[24]. C4bp protein circulates in mouse serum as a multimer of noncovalently linked α chains [25]. Protein levels are elevated in serum during the acute phase response [26], and males have higher serum levels than females (160 mg/l versus 60 mg/l) due to an effect of testosterone [27,28]. Expression of the murine C4bp α-chain mRNA has only been reported in the liver and in the epididymis [24,29,30]. As shown with human C4bp the mouse C4bp binds both mouse C4b *in vivo *and *in vitro *[25,27], and mouse C4 is unable to form a functional C3 convertase when bound to C4bp [27]. We recently reported the phenotype of the C4bp knockout mouse [31]. Serum from the mice had depressed C4 levels and increased hemolytic activity using antibody-coated sheep erythrocytes.

There are several potential mechanisms by which C4bp deficiency may modify disease progression in SLE. Reduced classical pathway regulation could enhance the ability to clear apoptotic cells, thereby reducing the supply of autoantigens. Similarly, an unregulated C3 convertase could generate more C3 for opsonization and clearance of immune complexes, thus limiting accumulation of these complexes in the kidney and other organs. Alternatively, local classical pathway dysregulation in the kidney could lead to increased inflammation and exacerbation of tissue damage.

To study the role of C4bp in SLE, we used a C4bp knockout mouse in an established experimental model. The MRL/lpr mouse is a spontaneous disease model for complement-associated inflammatory kidney disease, similar to lupus nephritis [32]. The *lpr *mutation, a retroviral transposon insertion in the FAS gene, results in loss of FAS function and thus a defect in FAS-mediated apoptosis [33]. When present on the MRL genetic background, the loss of FAS-mediated apoptosis results in massive lymphoproliferation with expansion of the B220^+^CD3^+^CD4^-^CD8^- ^cell population and the generation of autoreactive T cells [34]. The ensuing autoimmune disease is characterized by lymphadenopathy, complement activation, severe immune complex renal disease, and 50% lethality by 20–24 weeks of life [35]. We report here that C4bp deficiency does not modify disease severity in MRL/lpr mice.

## Materials and methods

### Mice

MRL/MpJ-Tnfrsf6^*lpr *^(Jackson Laboratories, Bar Harbor, ME, USA) and C4bp^-/-^C57BL/6 mice [31] were maintained in our animal colony. Backcrossing was performed using a speed congenics approach [36], and breeding of F3 mice was limited to those with >70% of screened loci encoding MRL alleles. Screening for MRL alleles of additional markers on chromosome 1 (D1Mit380 and D1Mit111) was performed to minimize the interval of 129 sequence surrounding the C4bp gene (67.6 cM on chromosome 1), as 129 alleles at multiple loci on this chromosome have been linked to enhanced autoantibody production [37]. After the third and sixth backcross, mice were bred to generate Fas^lpr/lpr^C4bp^-/- ^(KO MRL) mice, Fas^lpr/lpr^C4bp^+/- ^mice, and Fas^lpr/lpr^C4bp^+/+ ^(CTRL MRL) mice. Additional genotyping was not performed on the F6 mice but they were assumed >95% MRL genotype. These studies were reviewed and approved by the UTHSC-H Animal Welfare Committee.

### Immunophenotyping

Leukocytes were obtained from the spleens and axillary lymph nodes at 20 weeks of age. Cell populations were characterized with the following markers: CD3 (clone 145-2C11), CD4 (GK1.5), CD8 (53-6.7), CD25 (PC61.5), CD38 (90), CD19 (MB19-1), CD27 (LG.7F9), IgD (11–26c), CD11b (M1/70), and GR-1(Ly-6G) from eBiosciences (San Diego, CA, USA) and CD45R/B220 (RA3-6B2) and CD138 (281-2) from BD Pharmingen ( San Diego, CA, USA). A minimum of 10,000 events were collected and analyzed on a FACSCaliber using CellQuest software (BD Biosciences San Jose, CA, USA). Samples were obtained from five or six mice per group.

### Renal function

Timed urine collections were obtained from mice at 8, 12, 16, and 20 weeks of age. Urinary protein concentration was determined by BCA assay (Pierce, Rockford, IL, USA) and 24-hour excretion was normalized for body weight. Samples were measured in duplicate with 7–10 animals per group. Serum creatinine was measured by HPLC as previously described [38].

### Histologic analysis

Renal tissue was fixed in PBS-buffered 4% formalin, dehydrated and embedded in paraffin. Four-micron sections were stained with H & E or with periodic acid Schiff. Glomerular injury was graded in a blinded manner, with a minimum of 20 glomeruli scored per animal per group, as follows: the percentage of glomeruli containing cellular crescents, the percentage of glomeruli with sclerosis involving >25% of the glomerular tuft, and the degree of hypercellularity (0–3 scale).

Tubulointerstitial disease was graded on a 0–4 scale as follows: 0, no cellular infiltrates with back-to-back tubules, no evidence of fibrosis; 1, 0–5 cells per high-power field with minimal fibrosis; 2, 5–10 cells/high-power field with moderate fibrosis; and 3, >10 cells/high-power field with marked fibrosis.

Perivascular inflammation was graded on a 0–3 scale: 0, no cellular infiltrates surrounding branching arterioles or branching veins; 1, <10 cells; 2, <10 layers of cells; 3, >10 layers of cells.

### Immunostaining

OCT-embedded (optimal cutting temperature compound) snap-frozen 4 μm sections were stained with the following antibodies: FITC-conjugated goat anti-murine C3 (Cappel, Solon, OH, USA), FITC-conjugated goat anti-mouse IgG (Zymed/Invitrogen, Carlsbad, CA, USA), FITC-conjugated anti-mouse C1q (Cedarlane, Burlington, NC, USA), and rat anti-mouse C4 (Accurate, Westbury, NY, USA). For C4 staining, FITC-conjugated donkey anti-rat IgG was used for detection of primary antibody after absorbing for 15 min with normal mouse serum (Jackson ImmunoResearch, West Grove, PA, USA). Control staining was also performed using matched isotypes or IgG (data not shown).

Staining was quantified by incubation of sections with serial dilutions of antibody; endpoint titers were similar for all four antibodies between KO MRL mice and CTRL MRL mice. Staining was scored in a blinded manner on a relative scale of 0–3 using dilutions for each antibody on the linear portion of the titration curve.

### Autoantibody titers

Serum levels of antidouble-stranded DNA antibodies were measured by ELISA. Double-stranded DNA was derived by S1 nuclease (Boehringer/Roche, Indianapolis, IN, USA) treatment of calf thymus DNA (Rockland Gilbertsville, PA, USA). Wells were coated with 50 μg/ml poly-L-lysine overnight at 4°C, and then with 10 mg/ml double-stranded DNA at 37°C for 2 hours. After washing with PBS, sera were added in serial dilutions starting at 1/100 and incubated for 60 minutes at room temperature. After washing, horseradish peroxidase-conjugated goat anti-mouse IgG antibody or isotype-specific antibody (Jackson Immunoresearch) was added, followed by TMB (Pierce) for color development.

### Circulating immune complexes and serum complement assays

Blood was collected from the mice at the time of sacrifice and serum was prepared by clotting for 2 hours at 37°C followed by centrifugation. Circulating immune complex levels were determined by the C1q ELISA method previously described [39], with the following modifications. High protein binding plates (NUNC Maxisorp, Thermo Fisher Scientific) were coated with 1 μg/ml human C1q (AbD Serotec, Raleigh, NC, USA) in 0.1 M carbonate buffer (pH 9.6) for 48 hours at 4°C, and were then blocked for 2 hours at room temperature with 1% BSA in PBS. Serum samples were added in serial dilutions starting at a 1/50 dilution and plates were incubated for 2 hours. After washing with PBS 0.05% Tween-20, bound complexes were detected with horseradish peroxidase-conjugated goat anti-mouse IgG (BioRAD, Hercules, CA, USA). Color development was measured at 450 nm after incubation with TMB substrate (Pierce) and quenching with sulfuric acid. Mouse IgG was heat aggregated for 30 minutes at 37°C and was used as a positive control. Binding was measured in arbitrary units and normalized to binding of pooled normal mouse serum, used as a negative control (Jackson Immunoresearch).

The C3 and C4 levels in serum were measured by semiquantitative ELISA. Plates were coated with either goat anti-mouse C3 (Cappel) or rat anti-mouse C4 (Accurate) in carbonate buffer (pH 9.6) and were incubated overnight at 4°C. After washing and blocking with 5% BSA in PBS for 2 hours, sera were added in serial dilutions, starting at 1/100 and 1/10, respectively, and were incubated for 1 hour at room temperature. Bound protein was detected using horseradish peroxidase-conjugated goat anti-mouse C3 (Cappel) or rabbit anti-human C4c (Dako, Glostrup, Denmark) with horseradish peroxidase-conjugated donkey anti-rabbit IgG (Jackson Immunoresearch). Color development was measured at 450 nm after incubation with TMB substrate (Pierce) and quenching with sulfuric acid. Pooled normal mouse sera (Jackson Immunoresearch) was used as a positive control.

Classical pathway complement activity was measured by hemolytic assay. Sera were diluted in gelatin veronal buffer containing calcium and magnesium and were then added to IgM-sensitized sheep erythrocytes in 13 × 100 mm^2 ^glass test tubes (Complement Tech, Tyler, TX, USA). The percentage lysis at 37°C was determined after 1 hour. Reactions were stopped by adding ice-cold buffer and then removing cells by centrifugation at 3,000 rpm for 10 minutes at 4°C. Absorbance was read at 412 nm. Each serum sample was tested alone as a negative control, and incubation of sheep erythrocytes without serum was used to determine spontaneous lysis. One hundred percent lysis was defined as absorbance after incubation in hypo-osmolar buffer. The percentage lysis was calculated as follows:

Percentage lysis=OD412 (hemolytic test)−OD412 (negative control)OD412 (100% lysis)−OD412 (spontaneous lysis)×100
 MathType@MTEF@5@5@+=feaafiart1ev1aaatCvAUfKttLearuqqRPxAKvMB6bYrY9gDLn3AGiuraeXatLxBI9gBaebbnrfifHhDYfgasaacPi6xNi=xI8qiVKIOFjYdHaVhbbf9v8qqaqFr0xc9vqFj0dXdbba91qpepeI8k8fiI+fsY=rqGqVepae9pg0db9vqaiVgFr0xfr=xfr=xc9adbaqaaeGacaGaaiaabeqaaeqabiWaaaGcbaGaeeiuaaLaeeyzauMaeeOCaiNaee4yamMaeeyzauMaeeOBa4MaeeiDaqNaeeyyaeMaee4zaCMaeeyzauMaeeiiaaIaeeiBaWMaeeyEaKNaee4CamNaeeyAaKMaee4CamNaeyypa0tcfa4aaSaaaeaacqqGpbWtcqqGebarcqaI0aancqaIXaqmcqaIYaGmcqqGGaaicqGGOaakcqqGObaAcqqGLbqzcqqGTbqBcqqGVbWBcqqGSbaBcqqG5bqEcqqG0baDcqqGPbqAcqqGJbWycqqGGaaicqqG0baDcqqGLbqzcqqGZbWCcqqG0baDcqGGPaqkcqGHsislcqqGpbWtcqqGebarcqaI0aancqaIXaqmcqaIYaGmcqqGGaaicqGGOaakcqqGUbGBcqqGLbqzcqqGNbWzcqqGHbqycqqG0baDcqqGPbqAcqqG2bGDcqqGLbqzcqqGGaaicqqGJbWycqqGVbWBcqqGUbGBcqqG0baDcqqGYbGCcqqGVbWBcqqGSbaBcqGGPaqkaeaacqqGpbWtcqqGebarcqaI0aancqaIXaqmcqaIYaGmcqqGGaaicqGGOaakcqaIXaqmcqaIWaamcqaIWaamcqGGLaqjcqqGGaaicqqGSbaBcqqG5bqEcqqGZbWCcqqGPbqAcqqGZbWCcqGGPaqkcqGHsislcqqGpbWtcqqGebarcqaI0aancqaIXaqmcqaIYaGmcqqGGaaicqGGOaakcqqGZbWCcqqGWbaCcqqGVbWBcqqGUbGBcqqG0baDcqqGHbqycqqGUbGBcqqGLbqzcqqGVbWBcqqG1bqDcqqGZbWCcqqGGaaicqqGSbaBcqqG5bqEcqqGZbWCcqqGPbqAcqqGZbWCcqGGPaqkaaGaey41aqRaeGymaeJaeGimaaJaeGimaadaaa@B443@

C57BL/6 serum was used as a positive control.

### Statistics

The figures show the means, with error bars reflecting the standard error of the mean. A two-tailed unpaired Student's *t *test was used to test for significant differences between groups. The Mann–Whitney test was used to determine the significance of changes in histologic score and immunofluorescence data. Comparisons of serum C4 levels were analyzed by analysis of variance with a Bonferonni *P *value correction. Kaplan–Meier analysis was performed on survival curves using Prism software (GraphPad Software Inc., San Diego, CA, USA).

## Results

### Survival and lymphoproliferation

**Figure 1:**
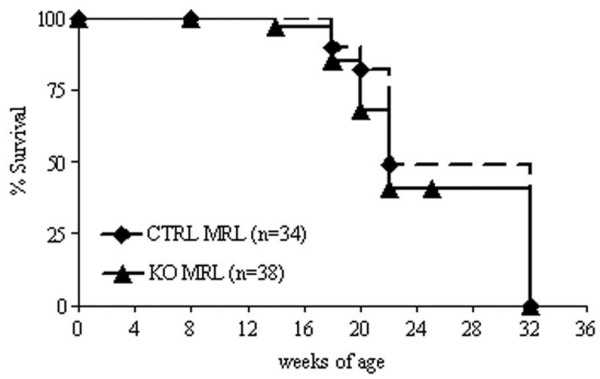
No difference in survival between knockout MRL mice and control MRL mice. C4bp^-/-^MRL/lpr (KO MRL) mice (solid line, *n *= 38) and littermate control (CTRL MRL) mice (dashed line, *n *= 34) from the F6 backcross were followed for up to 34 weeks. Mortality was quantified using Kaplan–Meier analysis. *P *= 0.15, KO MRL mice versus CTRL MRL mice (log-rank).

C4bp^-/-^C57BL/6 (KO B6) mice were back-crossed six generations onto the MRL genetic background. C4bp^+/- ^MRL mice were then intercrossed to obtain homozygous KO MRL mice and CTRL MRL control mice. These intercrosses resulted in the expected Mendelian ratios of homozygote and heterozygote progeny. MRL mice exhibit 50% mortality at 20 weeks of age [40]. Compared with CTRL MRL mice, the KO MRL mice had equivalent survival up to 34 weeks (Figure 1, 50% mortality 22 weeks). By 20 weeks, there was a significant reduction in body mass in KO MRL mice (39 ± 0.9 g) compared with CTRL MRL mice (43.3 ± 0.9 g, *P *< 0.005). Mice were sacrificed at this age for all further studies. Similar studies in F3 mice yielded an overlapping survival curve.

MRL mice develop massive lymphoproliferation with a preponderance of T cells in the lymph nodes and surrounding large vessels. There was a modest increase in the weight of axillary lymph nodes in KO MRL mice (798 ± 163 g) compared with CTRL MRL mice (502 ± 61 g, *P *< 0.05); however, there was no difference in splenomegaly (KO MRL mice, 702 ± 107 g; CTRL MRL mice, 791 ± 275 g; *P *> 0.05) or in the weight of the renal draining lymph nodes (KO MRL mice, 459 ± 113 g; CTRL MRL mice, 459 ± 118 g; *P *> 0.05). The KO MRL mice and CTRL MRL mice both developed large perivascular infiltrates in multiple organs including the lungs, the liver, the proximal small bowel, and the colon.

Detailed phenotypic analysis of lymphoid populations was performed. As expected, all MRL mice had expanded lymphocyte populations, primarily in CD4^-^CD8^- ^double-negative T cells. By flow cytometry, the absolute numbers of CD4^+ ^T cells, CD8^+ ^T cells, and CD4^-^CD8^- ^double-negative T cells in both the spleen and the lymph nodes in KO MRL mice were comparable with those in CTRL MRL mice (Table 1). To determine whether C4bp was important in B-cell responses in germinal centers, the proportions of IgD^+^CD27^- ^naïve B cells, CD27^+^CD38^+ ^centroblasts, CD27^+^CD38^- ^memory B cells, and IgD^-^CD138^+ ^plasma B cells were measured. There were no differences in these B-cell subsets between KO MRL mice and CTRL MRL mice.

**Table 1:** Splenic and lymph node T-cell and B-cell subsets

	C4bp knockout MRL mice (*n *= 5)	Littermate control MRL mice (*n *= 3)
CD4/CD8 ratio	0.42 ± 0.14	0.60 ± 0.34
Double-negative T-cell (%)	54 ± 4	49 ± 10
Naïve B cells (%)	49 ± 10	43 ± 10
Lymph node centroblasts (%)	29 ± 3	32 ± 4
Memory B cells (%)	6 ± 2	5 ± 0.01
Plasma B cells (%)	0.4 ± 0.2	0.3 ± 0.2

### Renal injury

MRL mice typically have chronic kidney disease characterized by proteinuria and renal insufficiency. Timed urine collections were performed in KO MRL mice and CTRL MRL mice at 8, 12, 16, and 20 weeks of age. Consistent with the model, there were age-dependent increases in protein excretion in both sets of mice; however, the degree of proteinuria was equivalent at all time points. At 20 weeks, KO MRL mice had a mean protein excretion of 0.53 ± 0.08 mg/g/day compared with 0.48 ± 0.05 mg/g/day in CTRL MRL mice (Table 2, *P *= 0.53). Moreover, KO MRL mice and CTRL MRL mice had abnormal elevations in serum creatinine, but the degree of elevation was only modestly lower in KO MRL mice (0.16 ± 0.03 mg/dl; CTRL MRL mice, 0.20 ± 0.04 mg/d; *P *= 0.39).

**Table 2:** Renal disease in C4bp knockout MRL mice compared with littermate control MRL mice

	C4bp knockout MRL mice (*n *= 16)	Littermate control MRL mice (*n *= 13)
Proteinuria (mg/g/day)	0.53 ± 0.08	0.48 ± 0.05
Serum creatinine (mg/dl)	0.16 ± 0.03	0.20 ± 0.04
Hypercellularity	1.9 ± 0.2	2.0 ± 0.3
Crescents (%)	9 ± 4	11 ± 6
Sclerosis (%)	17 ± 7	38 ± 9
Periglomerular leukocytes	14 ± 3	15 ± 2
Tubulointerstitial disease	1.4 ± 0.3	1.3 ± 0.3
Vasculitis	1.3 ± 0.1*	2.4 ± 0.2
IgG staining	2.0 ± 0.2	2.0 ± 0.2
C3 staining	1.5 ± 0.2	1.6 ± 0.3
C4 staining	1.3 ± 0.2	1.3 ± 0.1
C1q staining	0.6 ± 0.1	0.8 ± 0.1

*P < 0.0001.

**Figure 2:**
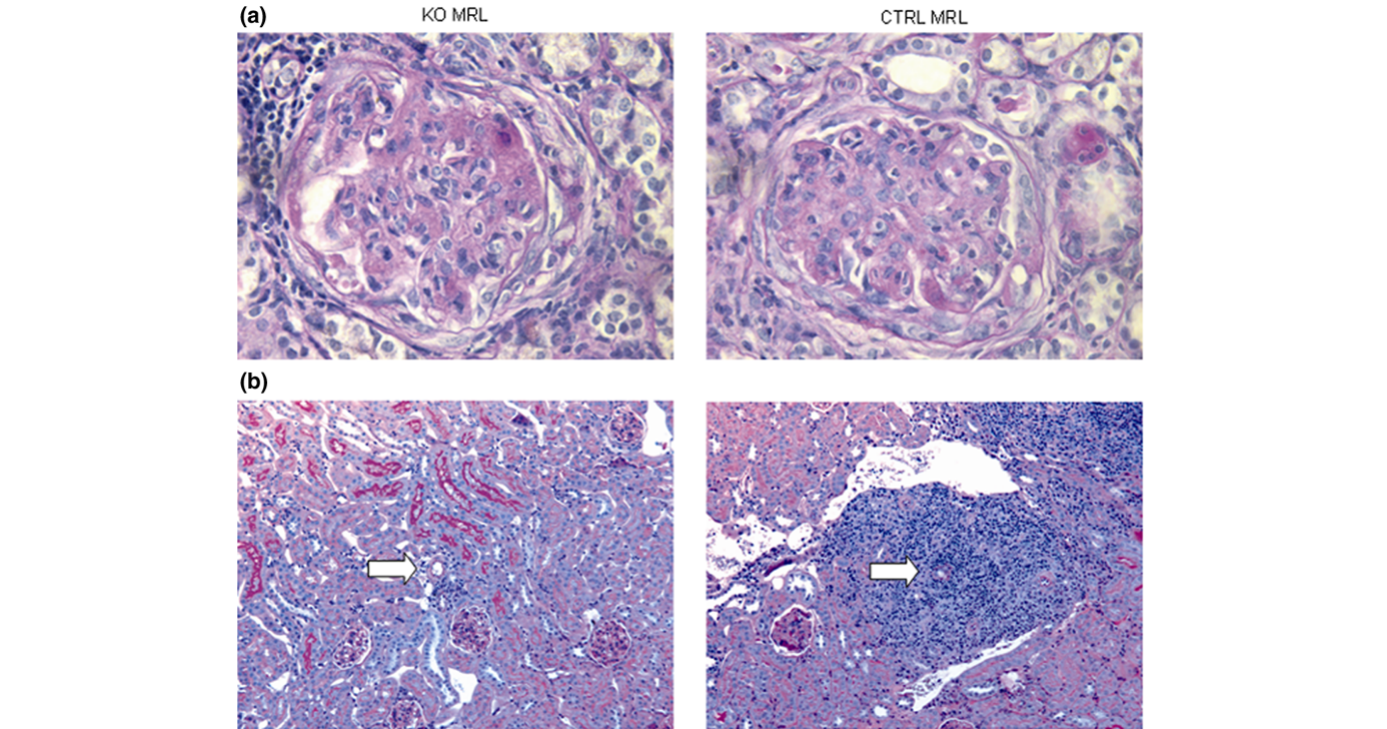
Renal histopathology in knockout MRL mice and control MRL mice. Sections showing the renal histopathology of C4bp^-/-^MRL/lpr(KO MRL) mice and littermate control (CTRL MRL) mice. **(a) **Representative formalin-fixed sections from the kidney stained with periodic acid Schiff (0.75NA, 400× magnification). Glomeruli with crescentic changes are shown. **(b) **Sections stained with periodic acid Schiff showing perivascular inflammation around branching arteries (white arrows) (0.15NA, 50× magnification).

Histologically, KO MRL mice and CTRL MRL mice had proliferative glomerulonephritis, tubulointerstitial inflammation with fibrosis, and large perivascular infiltrates (Figure 2). There were equivalent degrees of glomerular hypercellularity and similar proportions of glomerular crescents. Scoring revealed a modest decrease in glomerulosclerosis in KO MRL mice (Table 2) but there was large variability between mice, which impacted the statistical significance (*P *= 0.09). Histologic scores for tubulointerstitial disease and periglomerular leukocyte accumulation (*P *= 0.87 and *P *= 0.78, respectively) were identical in KO MRL mice and CTRL MRL mice (Table 2). There was a two-fold decrease in the perivascular leukocyte number in KO MRL mice kidneys (*P *< 0.0001; Figure 2). Scoring of kidney pathology was performed at 20 weeks on all female mice; however, sampling mice at other ages showed that the progression of disease in both genders was equivalent between strains.

The pathogenesis of glomerular disease in the MRL mouse involves immune complex accumulation with deposition of circulating complement proteins as well as increased localized complement production. Immunofluorescent antibody staining showed large amounts of both complement protein C3 and IgG in the glomerular mesangium and in the capillary loops (data not shown). There were no differences in the degree of staining in KO MRL mice and CTRL MRL mice as measured by serial dilution of antibody or by scoring of representative glomeruli by blinded observers (*P *= 0.55 and *P *= 1.0, respectively; Table 2). The degree of local complement activation via the classical pathway was also assessed in the kidney by immunostaining. The KO MRL mice kidneys and the CTRL MRL mice kidneys had similar degrees of C1q and C4 (*P *= 0.55 and *P *= 1.0, respectively; Table 2). There were also no differences in complement and IgG staining at earlier time points. Therefore, there appeared to be no differences in renal handling of immune complexes by KO MRL or CTRL MRL mice.

### Systemic immune responses

**Figure 3:**
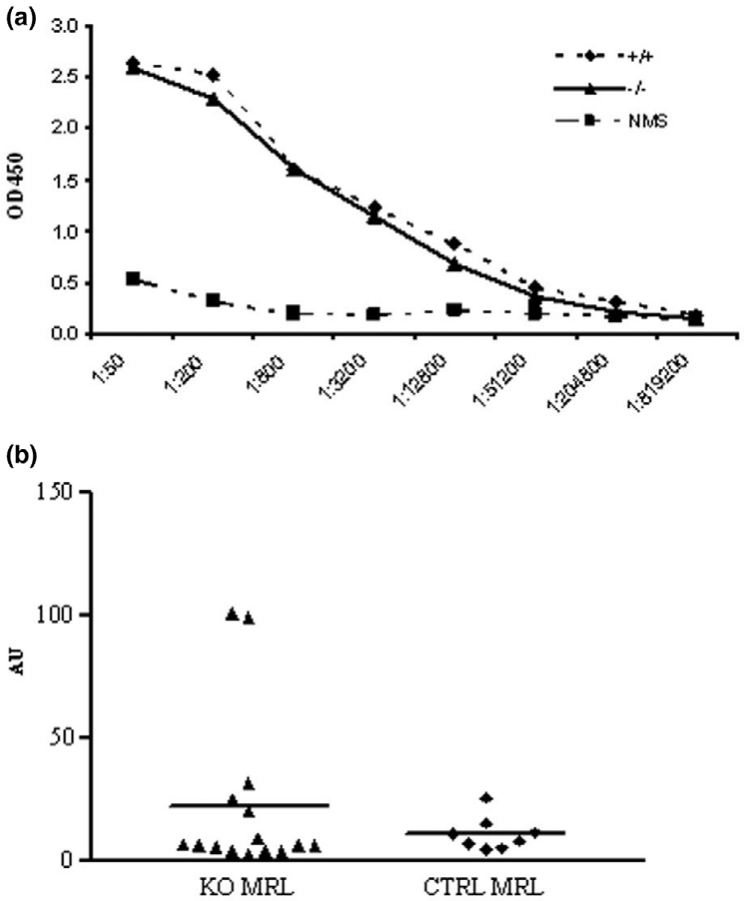
Similar serum autoantibody titers and circulating immune complexes in knockout MRL and control MRL mice. **(a) **Sera from C4bp^-/-^MRL/lpr (KO MRL) mice (▲, solid line, *n *= 15) and littermate control (CTRL MRL) mice (◆, dashed line, *n *= 8) were tested for binding to double-stranded DNA by ELISA using serial serum dilution (*x *axis). Pooled normal mouse serum from nonautoimmune mice (■, NMS) was used as a negative control. *P *> 0.05, KO MRL mice versus CTRL MRL mice. OD450, optical density at 450 nm. **(b) **Sera from 20-week-old KO MRL mice (▲, *n *= 15) and littermate control mice (◆, CTRL MRL, *n *= 8) were tested for binding to human C1q by ELISA. Data for each individual mouse are shown and mean increases in immune complex levels are displayed as solid lines. *P *> 0.05, KO MRL mice versus CTRL MRL mice. AU, arbitrary units.

MRL mice have lymphoproliferation and autoantibody production due to loss of tolerance. KO MRL mice and CTRL MRL mice both had elevated antidouble-stranded DNA antibody titers by 20 weeks of age compared with pooled serum from nonautoimmune mice (Figure 3, endpoint titer 1:204,800 in both KO MRL and CTRL MRL mice sera). Moreover, there were no differences in titers of the IgG_1 _(Th2-predominant) or IgG_2a _(Th1-predominant) autoantibody subsets (endpoint titers 1:51,200 and 1:204,800, respectively).

As a consequence of high titers of autoantibodies, MRL mice have increased production of antibody–antigen immune complex in the circulation. These immune complexes are cleared by the reticuloendothelial system, in part due to opsonization and solubilization by complement proteins. To determine whether C4bp knockout mice had an altered ability to clear immune complex due to impaired classical pathway complement regulation, we measured immune complex levels in the serum of 20-week-old mice. KO MRL mice serum and CTRL MRL mice serum had significantly more immune complex than normal mouse sera. There was no difference in immune complex levels in KO MRL mice compared with CTRL MRL mice (*P *= 0.36; Figure 3b), although the two mice with the largest burdens of circulating immune complexes were KO MRL mice.

**Figure 4:**
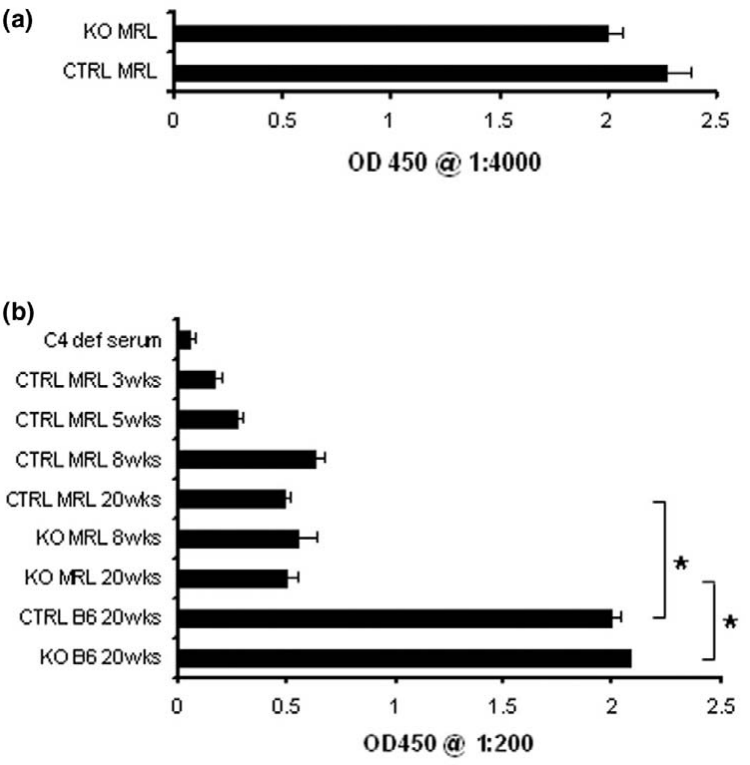
Serum C3 and C4 levels in knockout MRL mice, control MRL mice, and nonautoimmune mice. **(a) **Serum C3 levels from 20-week-old C4bp^-/-^MRL/lpr (KO MRL) mice (*n *= 6) and littermate control (CTRL MRL) mice (*n *= 5) were measured by ELISA, and means values for 1:4,000 dilution are shown. *P *> 0.05 at all dilutions tested. OD450, optical density at 450 nm. **(b) **Serum C4 levels from 20-week-old KO MRL mice (*n *= 13) and CTRL MRL mice (*n *= 9) were compared with levels from mice at different ages as well as 20-week-old KO C57BL/6 (B6) mice, CTRL B6 mice, and C4-deficient B6 mice (*n *= 3 for each). Serum levels were measured by serial dilutions using sandwich ELISA, and mean values for 1:200 dilution are shown. *P < 0.001.

To determine whether modest increases in circulating immune complex levels could be explained by relative decreases of classical pathway complement proteins in the circulation of C4bp knockout mice, C3 and C4 levels were measured by semiquantitative ELISA. At 20 weeks of age, both C3 and C4 levels in KO MRL mice were equivalent to levels in CTRL MRL serum (Figure 4). C4 levels were also equivalent at 8 weeks of age. Of note, the levels of C4 in CTRL MRL mouse serum were 16-fold lower than those measured in wildtype CTRL B6 mice or in KO B6 mice (*P *< 0.001). In CTRL MRL mice, the serum C4 levels rise four-fold from 3 weeks of age to 8 weeks of age and then remain unchanged until at least 20 weeks of age. To confirm that there was no difference in basal classical pathway activity in serum from the KO MRL mice compared with that of CTRL MRL mice, complement hemolytic assays were performed. There was no measurable difference between KO MRL mice and CTRL MRL mice (*P *= 0.11), but the activity of KO MRL and CTRL MRL sera was significantly less than that of KO B6 serum or CTRL B6 serum (*P *< 0.001; data not shown).

## Discussion

We report the phenotype of C4bp-deficient MRL mice. Given that the MRL mouse has long been held as a murine model of the immune complex renal injury seen in patients with lupus nephritis, it was surprising that mice lacking the critical regulator of the classical pathway of complement activation had no differences in mortality or morbidity compared with C4bp-sufficient littermate control mice. There were no significant differences in the severity of renal injury between strains with respect to the glomerular deposition of complement proteins or immunoglobulins. Similarly there were no differences in either the degree of glomerular proliferation, of periglomerular inflammation, or of tubulointerstitial disease. In addition there was no evidence of increased complement activation either locally within the kidney or systemically in KO MRL mice at any age. C4bp, and thus negative regulation of the classical pathway of complement activation, therefore appears to play a minimal role in modulating disease severity in the MRL mouse.

There are several possible explanations for the lack of phenotypic differences between the C4bp-deficient mice and the control mice. First, it is possible that the classical pathway is maximally activated in the setting of autoimmunity in the MRL mouse, and that genetic targeting of C4bp does not increase the classical pathway hemolytic activity as it does in nonautoimmune mice (Soimo and Wetsel, manuscript in preparation). To investigate this possibility, serum C4 levels were measured by ELISA at various ages. The data indicate that, similar to humans, serum C4 levels rise early in life from 3 weeks to 8 weeks of age. After 8 weeks, the levels remain constant until at least 20 weeks, when kidney disease becomes evident. Interestingly, in comparison with CTRL MRL mice, CTRL B6 mice had significantly higher hemolytic activity and serum C4 levels. It is unlikely that these findings are due to complement consumption mediated by either tissue deposition or circulating immune complexes in the MRL mouse, as at 8 weeks of age, prior to the onset of overt injury, serum C3 levels were similar between the two strains (data not shown). It would therefore appear that, with respect to CTRL B6 mice, CTRL MRL mice have C4 deficiency marked by functional reductions in classical pathway hemolytic activity.

Two C4 genes map to the H-2 region of mouse chromosome 17. The MRL strain is H-2^k ^and encodes only one C4^k ^allele, which is aberrantly spliced in hepatocytes [41]. An intronic insertion encodes an alternative 5' splice site, resulting in an inframe stop codon in the mRNA and a truncated C4 protein that is not secreted [42]. Nonhepatic tissues do not utilize this splice site, and they express a full-length mRNA and a wildtype protein [43,44]. As the majority of C4 in the serum is derived from the liver, mice with the H-2^k ^haplotype express 10-fold to 20-fold lower amounts of C4. C57BL/6 mice are H-2^b ^and encode C4 and a related protein Slp (sex-limited protein). The C4^b ^allele lacks the intronic insertion in C4^k ^and is expressed at higher levels, as we have confirmed. In addition, Slp is expressed in high levels in male mice. These differences are likely to explain the decreased hemolytic activity in MRL serum compared with C57BL/6 serum. MRL mice and BXSB mice, both mouse models for lupus-like disease, have previously been described to have lower C4 levels than B6 mice [45]. The H-2 region maps to qualitative trait loci *Sle4 *and *Lbw1*, both identified by genetic mapping in mouse models of SLE [46,47]. It therefore seems more probable that the development of autoimmunity in MRL mice is in part related to a functional deficiency in C4, similar to that seen in humans with deficiencies in early classical pathway components such as C1q, C2, and C4.

As the local, nonhepatic, synthesis of C4 is normal in mice with the H-2^k ^allele, a second explanation for the lack of phenotype in C4bp KO MRL mice is that the classical pathway plays only a minor role in local complement-dependent injury in the MRL mouse. C3 deposition in the kidney was much more intense than C4 and C1q deposition, and this may be reflective of a larger role for the alternative pathway in cleavage and deposition of C3. The alternative pathway requires factor B to form the C3 convertase, and in the MRL background factor B knockout mice have less proteinuria, decreased renal pathology scores, less glomerular IgG staining, and less renal vasculitis [48]. Recent studies in a pure immune complex model of renal injury additionally conclusively demonstrated that the renal injury seen in this model was alternative pathway dependent [49]. Alternatively, it is possible that the lack of differences in local complement deposition and subsequent renal injury may also be reflective of the relative contribution of fluid phase regulatory proteins, such as C4bp, versus regulatory proteins expressed on the cell surface, such as MCP and DAF. Our data combined with those reported in the factor-B-deficient MRL mouse, however, strongly support the hypothesis that the principal pathway that drives complement-dependent renal injury in the MRL mouse is the alternative pathway.

In addition to the primary role of C4BP in negatively regulating classical pathway activation, C4bp has been proposed, either directly or indirectly, to modulate a variety of biologic processes including hemostasis, B-cell activation, and immune complex clearance. With respect to hemostasis, murine C4bp lacks the β chain present in human C4bp, and thus is unable to bind protein S. C4bp therefore plays no role in the mouse system in regulating the coagulation cascade. It has recently been reported that the α chain of C4bp has a functional role in mediating B-cell proliferation and class switching via its interactions with the CD40-CD40 ligand system. While this interaction was not directly examined in the current report, there were no differences in either absolute B-cell number, serum levels of autoantibodies, or subclasses of antidouble-stranded DNA antibodies between C4bp-sufficient mice or C4bp-deficient mice. In the context of the MRL mouse, therefore, it appears that C4BP plays no role in the regulation of B-cell responses. As an intact classical pathway is required for proper clearance of immune complexes and apoptotic bodies, C4bp as a negative regulator of the classical pathway should impact clearance of immune complex by limiting the activity of the classical pathway C3 convertase, and subsequent generation of C3b needed for solubilization of immune complexes. We were unable, however, to demonstrate any difference in circulating immune complex between the C4bp-sufficient mice and C4bp-deficient mice. Although it is possible that the reduced levels of C4 in the MRL strain limit the intrinsic capacity of the classical pathway to generate C3b, there are data to suggest that amplification of C3b generation via the alternative pathway is required for immune complex clearance [50]. This is believed to be due to the inefficiency of C3b binding to the immune complex: only 10% of generated C3b binds to the complex. Loss of the negative regulator the classical pathway therefore appears to have minimal impact on immune complex processing when the Alternative Pathway is intact. Further study of immune complex and apoptotic cell clearance in C4BP and factor B knockout mice in a C4-sufficient genetic background could confirm the relative importance of these two pathways in immune complex clearance.

One notable finding in C4bp knockout mice kidneys was their small perivascular infiltrates compared with very large infiltrates seen in control mice. This finding was tissue specific, as there were no differences in perivascular infiltrates in other tissues. The biology of C4 and its cleavage products in the mouse is unclear due to a paucity of reagents available for this animal. It is possible that local production of C4 in the kidney is more responsible for leukocyte accumulation in this than in other organs. C4bp may be required for optimal cell recruitment, perhaps due to binding of a chemotactic product of C4b cleavage. Alternatively, C4bp may modulate kidney endothelial cell function in a complement-independent manner. Nonetheless, differences in perivascular leukocyte accumulation in renal vessels did not correlate with other histologic parameters, with kidney function, or with survival.

## Conclusion

In summary, the current studies in C4bp-deficient mice fail to demonstrate any significant impact on survival or disease severity in the MRL mouse model of lupus nephritis. Furthermore, this lack of impact on disease phenotype appears to be due to a relative deficiency of C4 in the MRL mouse strain that results in a functional reduction in the classical pathway hemolytic activity. Given previous data showing that renal injury in the MRL mouse is independent of Fc receptors [51], our studies showing the functional deficiency of C4 in these mice, and that the loss of the major negative regulator of the classical complement pathway fails to impact disease severity, the use of the MRL mouse as a prototypical model of immune complex renal injury may need to be reconsidered. Alternatively, new mechanisms for immune-complex-mediated renal injury need to be considered.

## Abbreviations

B6 = C57BL/6; BSA = bovine serum albumin; C4bp = C4 binding protein; CTRL = control; ELISA = enzyme-linked immunosorbent assay; Fc = crystallizable fragment; H & E = hematoxylin and eosin; HPLC = high-performance liquid chromatography; KO = knockout; MRL = MRL/MpJ-Tnfrsf6^lpr^; PBS = phosphate-buffered saline; SLE = systemic lupus erythematosus.

## Competing interests

The authors declare that they have no competing interests.

## Authors' contributions

SEW planned and performed the majority of the experiments and was primary author of the manuscript. KS performed the hemolytic assays and assisted in interpreting the data.

RAW generated the knockout mice, assisted in interpreting the data, and critically reviewed the manuscript. MCB acquired funding, planned and supervised the experiments, and revised and edited the manuscript. All authors read and approved the final manuscript.
